# Subjective social status and inequalities in depressive symptoms: a gender-specific decomposition analysis for South Africa

**DOI:** 10.1186/s12939-019-0996-0

**Published:** 2019-06-13

**Authors:** Chipo Mutyambizi, Frederik Booysen, Per Stornes, Terje A. Eikemo

**Affiliations:** 1 Research Use and Impact Assessment (RIA), Human Sciences Research Council (HSRC), HSRC Building, 134 Pretorius Street, Pretoria, 0002 South Africa; 2 School of Economic and Business Sciences (SEBS), University of the Witwatersrand (Wits), Johannesburg, South Africa; 3 Centre for Global Health Inequalities Research (CHAIN), Department of Sociology and Political Science, Norwegian University of Science and Technology (NTNU), Trondheim, Norway

**Keywords:** Subjective social status, Depression, Inequality, Concentration index, Gender, Decomposition analysis, South Africa

## Abstract

**Background:**

Inequalities in mental health are a notable and well documented policy concern in many countries, including South Africa. Individuals’ perception of their position in the social hierarchy is strongly and negatively related to their mental health, whilst the global burden of poor mental health is greater amongst women. This paper offers a first glimpse of the factors that shape gender-based health inequalities across subjective social status.

**Methods:**

This study employs the cross-sectional 2014 South African Social Attitudes Survey (SASAS). The prevalence of depressive symptoms is measured with the aid of the CES-D 8-item scale, with analyses disaggregated by gender. Concentration indices (CI) are used to measure inequalities in depressive symptoms related to subjective social status. The study applies the Wagstaff decomposition to determine the factors that contribute to these gender-based inequalities.

**Results:**

More than 26% of the study sample had depressive symptoms (95% CI 24.92–28.07). The prevalence of depressive symptoms is significantly more pronounced in females (28.46% versus 24.38%; *p* = 0.011). The concentration index for depressive symptoms is − 0.276 (95% CI -0.341 – − 0.211), showing large inequalities across subjective social status. The observed SSS-related inequality in depressive symptoms however is higher for males (CI = -0.304) when compared to females (CI = -0.240) (*p* = 0.056). The most important contributor to SSS-related inequalities in depressive symptoms, at 61%, is subjective social status itself (contributing 82% in females versus 44% in males). Other variables that make large contributions to the inequalities in depressive symptoms at 11% each are race (contributing 2% in females versus 25% in males) and childhood conflict (contributing 17% in females versus 4% in males).

**Conclusion:**

Policy makers should target a reduction in the positive contribution of SSS to depression via the implementation of programmes that improve social welfare. Given the much greater contribution to inequalities among females, these policies should target women. Policies that protect children and especially the girl child from conflict can also be useful in reducing inequalities in depression related to subjective social status during adulthood. Overall, there is need for a multi-sectoral approach to address these inequalities.

## Background

Research synthesis provides compelling evidence of the positive and reciprocal association between poor mental health and poverty or low socioeconomic status [1–3]. There is also robust empirical evidence from research synthesis that subjective social status (SSS), or one’s perceived position in the social hierarchy, is strongly related to health, with low SSS being associated with poorer health outcomes [4–6]. This SSS gradient is particularly evident in the case of mental health [7–18]. The one explanation put forward for SSS being an important predictor of health outcomes is that it represents a cognitive average of multiple markers of socioeconomic status (SES) that accounts for past, current and future prospects and overall life chances [7, 19]. The second explanation is that the SSS gradient embodies the hierarchy-health relationship, which hypothesises that relative social position has a direct and indirect impact on health. SSS has a direct effect on physiological processes and neuroanatomic structures, which enhances humans’ biological vulnerability to disease. In addition, SSS indirectly affects health via an exposure to unhealthy behaviours [7]. Unlike OSS that mainly captures the mechanism of material deprivation, SSS also encapsulates the psychological mechanism of stress responsible for the socioeconomic health gradient [19]. Consequently, the SSS gradient in mental health generally persists when adjusting results for objective measures of socioeconomic status (OSS) such as education, occupation and income [7–11, 15–18].

The study of these SSS gradients in health with the aid of standard inequality indices such as the relative index of inequality (RII) [11–13] and the concentration index [20, 21] is limited to developed countries in Europe and the United Kingdom and United States. Furthermore, the predictors of SSS-related inequalities in health have not been investigated with the aid of decomposition analysis, which is important from a policy perspective insofar as it allows the identification of the factors driving these inequalities. Nor has such analysis been conducted from a gender-based perspective, which is necessary because the global burden of poor mental health is more pronounced in women [22–24]. In fact, gender-specific analysis on SSS and mental health has been restricted to developed countries contexts such as the United Kingdom [7, 14], Sweden [13] and Finland [18]. Such gender-specific analysis also meets the recent call for gender-based reporting in health inequality research [25]. Adverse childhood experiences, moreover, which are strongly associated with mental health in adulthood [26], has only been investigated in a single UK study on mental health and SSS [7] and then only childhood SES.

The inclusion in the nationally representative South African Social Attitudes Survey (SASAS) of the European Social Survey’s (ESS) theoretically and methodologically grounded module on the social determinants of health [27], which includes information on childhood adversity of an economic and psychological nature, allows a first glimpse of the gender-specific factors that shape SSS-related inequalities in mental health in a developing country context. The study’s setting, moreover, is characterised by a high burden of depression [28–32], especially among women [30, 31, 33, 34], with depressive disorders being the fifth leading cause of years lived with disability by South Africans according to the 2017 Global Burden of Disease study [35]. South Africa is also characterised by high and rising social and economic inequality [36] that is the remnant of the colonial and apartheid systems’ discriminatory policies and their influence on the country’s social landscape and the population’s psyche. Yet, only a single study has investigated the link between SSS and mental health in South Africa [34], but with a small purposive sample rather than a nationally representative survey. The questions posed in this paper are also important from a global policy perspective given the objectives of Sustainable Development Goals 3 and 10 to respectively reduce the burden of mental health and of inequalities by 2030 [37].

## Methods

### Data

The data used in the study originates from the nationally representative 2014 South African Social Attitudes Survey (SASAS), which fielded the survey module on social determinants of health in the European Social Survey (ESS7) in a developing country context [27]. This survey module on the social determinants of health has been extensively used to document health inequalities in European countries [38–40], including disparities in mental health [41–43], emphasising its suitability for this study. SASAS 2014 received ethical clearance from the HSRC and face-to-face interviews were conducted with a cross-sectional sample of 3108 consenting individuals aged 16 years and older. All study participants provided informed consent. The study used a two-stage stratified sampling design and the response rate was 89% [44].

### Measures

#### Health outcomes

The depression scale of the Centre for Epidemiologic Studies (CES-D) is widely used and has acceptable screening accuracy in general populations [45]. The 2014 SASAS adult questionnaire included the CES-D 8 variant of the scale, which contained a set of eight items that asked respondents if: they felt depressed, everything was an effort, sleep was restless, felt lonely, felt sad, could not get going, were happy or enjoyed life during the past week. Responses were none, some, most or all of the time, scored 0–3. The CES-D 8 is a widely used and reliable measure of depression symptomology that has been applied both internationally and in South African studies [30, 41]. Previous studies have shown that the CES-D-8 scale is valid and reliable [46, 47]. In terms of internal consistency, the Cronbach’ alpha for the eight items in the scale is 0.773. Approximately 96% of the study sample responded to all eight questions. Following Van dem Knesebeck et al. [48] and Huijts et al. [41], we created a 24 point scale from the individual responses to the eight questions [41]. Positive effect questions (such as happiness) were reverse scored. Mean imputation was applied where respondents had missing answers on 1 to 4 of the 8 questions (3.7%). We then used the scale to create a dichotomous variable that took on the value of 0 when the scale had values 0 to 9 and 1 when the scale had values 10 to 24 [41, 48]. This outcome henceforth is referred to as ‘depressive symptoms’.

#### Subjective social status

This study uses the measure of SSS to investigate the inequality in depressive symptoms. The construct validity of this scale is demonstrated by Cundiff et al. [49]. Similar to other studies [4, 7, 13, 14, 50] and using a scale running from 1 (bottom) to 10 (top), individuals were asked to position themselves on this social hierarchy by the instruction: “In our society, there are groups which tend to be towards the top and groups which tend to be towards the bottom. Below is a scale that runs from the top (scoring 10) to the bottom (scoring 1). Where would you put yourself on this scale”? This variable was included in our study in two versions: one as a continuous variable for the construction of the CI, using the original scoring, and one as a categorical variable for the subsequent decomposition of inequalities in the health outcomes. Values 1&2 = 1, 3&4 = 2, 5&6 = 3, 7&8 = 4, 9&10 = 5.

#### Socio-demographic characteristics

The socio-demographic variables included in our analysis are gender, race, age, marital status and place of residence. For accurate reporting and to emphasize the social and cultural differences between men and women we make use of the term gender instead of sex [51]. Gender was included as a binary variable: male (=0) and female (=1). Race was included as a categorical variable: African Coloured and Indian/Asian/White. Age was measured in years and included as a categorical variable with the following groups: 18–34 years, 35–64 years, and 65 + years. Marital status was included as a binary variable: married/civil partnership (=0) and single (=1). Place of residence was categorised as urban formal, urban informal, tribal, and rural formal.

#### Other variables

We also included a range of other socio-economic variables, measures of childhood adversity and lifestyle factors which influence health [52, 53]. The socio-economic variables included were employment status and education. Employment was coded in three categories: employed, non-labour force participant and unemployed. Education was coded in five categories of no education, primary, secondary, matric, and tertiary education.

Previous literature also reports that those who experience adverse childhood conditions are more likely to experience mental disorders [26, 54]. Survey respondents were asked the following two questions regarding childhood adverse experiences: (1) Please tell me how often you and your family experienced severe financial difficulties when you were growing up? (2) Please tell me how often there was serious conflict between the people living in your household when you were growing up? Based on this detail, the childhood adversity variables included in our study are described as past childhood financial difficulties and past childhood conflict. Both were included as binary variables: hardly/never (no = 0) and always/sometimes (yes = 1). In order to focus on the effects of these adversities during adulthood, respondents under 18 years were excluded from the analysis (*n* = 47).

Lifestyle factors included in the analysis comprise fruit consumption, vegetable consumption, physical activity, smoking, and alcohol consumption. Previous research indicates that healthier diets (more fruits and vegetables) are associated with better moods and less depression [55]. Fruit and vegetable consumption were both included as binary variables that took on the following values: 0 - when consumed less than once a day over a week and 1 - when consumed at least once a day. Respondents were asked how many of the last 7 days did they walk quickly, do sports or other physical activity for 30 min or longer. This was included as a binary variable; 0 - exercised up to 2 days a week and 1 - exercised at least 3 days a week. Respondents were also asked to describe their smoking behaviour. This was included as a binary variable with 0 - less than daily/never and 1 - daily. Alcohol consumption was also included as a binary variable with 0 - less than or once a week/never and 1 - more than once a week.

#### Measuring inequalities in depressive symptoms related to subjective social status

Various measures have been proposed for measuring inequalities in health. Wagstaff et al. (1991) provides three basic criteria that an index of inequality in health needs to satisfy. These are (1) the measure should reflect the experiences of the entire population and not just the extreme ends (2) the measure should be sensitive to changes in the population in socioeconomic groups (3) the measure should reflect the socioeconomic elements to health inequalities [56]. The widely employed concentration index (CI) satisfies these minimum requirements [56] and is applied in this paper as a measure of inequality.

The companion concentration curve (CC) plots the cumulative percentage of depressive symptoms on the vertical axis against the cumulative percentage of the sample ranked by SSS starting with those who reported lowest SSS and ending with those who reported higher SSS on the horizontal axis [57]. If everyone had the same depressive symptoms irrespective of their SSS then the CC would like on the 45-degree line, also known as the line of equality. If the depressive symptoms are more concentrated amongst those with low (high) SSS the curve lies above (below) the 45-degree line [57].

The CI is derived from the concentration curve (CC) and lies between the values of − 1 and + 1 [57]. The index takes on a value of zero if there are no inequalities in the outcome variable (in this case depressive symptoms) and takes on negative values if depressive symptoms are more concentrated amongst those of low social status and positive values if depressive symptoms are concentrated amongst those of high social status [57]. The CI can then be measured as twice the covariance of the health variable and the ranking of the living standards variable r (in this case SSS), divided by the mean of the health measure (*μ*):

1 $$ CI=\frac{2}{\mu}\mathit{\operatorname{cov}}\left(h,r\right) $$

Since the depressive symptoms variable in our study is binary we also make use of the Erreygers corrected concentration index which is algebraically expressed as shown below [58].

2 $$ E(h)=8\operatorname{cov}\left({h}_i,{R}_i\right) $$

Our study makes use of the *conindex* command in STATA to estimate SSS-related inequalities in depressive symptoms [59].

#### Decomposition of the CI

The CIs for depressive symptoms can be decomposed to show the contributions of individual factors to SSS-related inequalities. Our study makes use of the method proposed by Wagstaff et al. [60], which uses the following linear equation.

3 $$ {h}_i=\alpha +\sum \limits_{j=1}^q{\beta}_j{x}_{ij}+{\varepsilon}_i $$

Where h is the health variable of individual i, α is the constant, x is a set of variables such as demographic and socio economic factors, ε is the error term. If we have such linear model as shown in eq. (3), Wagstaff et al. shows that the concentration index for h_I_ can be written as [60]:

4 $$ CI(h)=\sum \limits_{j=1}^q\frac{\beta_j\overline{x_j}}{\mu_h} CI\left({x}_j\right)+\frac{GC_{\varepsilon }}{\mu_h} $$

This equation allows us to calculate the contribution of each factor to SSS-related inequalities in depressive symptoms. Since we applied the Erreygers normalisation to the calculation of the CI for the SSS-related inequalities in depressive symptoms, the corrected CI for the depressive symptoms variable is formulated as:

5 $$ E(h)=4\sum \limits_{j=1}^q{\beta}_j\overline{x_j} CI\left({x}_j\right)+4{GC}_{\varepsilon } $$

Since the Generalised Linear Models (GLM) (with binomial family and identity link) is reported to be the best choice when decomposing inequality in a binary variable [61], eq. (5) is estimated using GLM and used to decompose SSS-related inequalities in depressive symptoms.

The contribution made by each factor is dependent on the sign and size of the calculated elasticity and CI for each regressor. If the contribution of variable *X* is positive, then inequality in depressive symptoms would decrease if variable *X* becomes equally distributed across the social group, ceteris paribus. The opposite is also true, i.e. if a factor’s contribution is negative, the absence of inequalities in that variable would result in an increase in inequality, ceteris paribus. We also apply a bootstrapping method using 500 replications to estimate standard errors for the absolute contributions.

Statistical analysis was performed in STATA software version 13. Our data was weighted with post-stratification weights in order to account for clustering and survey design effects. The analysis reported here was restricted to individuals above the age of 18 years and those with non-missing information on subjective social status. Among the cross-sectional sample of 3108 consenting individuals aged 16 years and older, 47 were below the age of 18 and were excluded. A further 34 had missing information on subjective social status and were also excluded. Our final analytic sample was 3027.

## Results

### Descriptive statistics

Table 1 shows the study population’s characteristics. About half of the respondents were male (48%) whilst 52% were female. Most of the participants were African (78%), between the ages of 18 to 34 years (49%), single (60%), and resided in formal urban settlements (67%). The overall mean of the CES-D 8 scale was 7.39 (SD – 4), whilst the CES-D 8 score for females (7.72) was statistically significantly larger than for males (7.04) (*p* < 0.001). Approximately 26% of the population had depressive symptoms that could be indicative of clinical depression. The prevalence of depressive symptoms was statistically significantly higher amongst women (28.46%) than men (24.38%) (*p* = 0.011).

**Table 1:** Summary statistics, by gender

Variable	Male	Female	Total
CES-D 8 scale			
Mean	7.04	7.72	7.39
Standard deviation (SD)	4.09	4.00	4.06
Median	7.00	7.16	7.00
P25	4.00	5.00	5.00
P75	9.00	10.00	10.00
Depressive symptoms (%)	24.38	28.46	26.49
Gender			
Male			48.32
Female			51.68
Race			
African	77.24	78.79	78.04
Coloured	9.34	9.22	9.28
Indian/Asian/White	13.42	12.00	12.68
Age			
18to34	52.04	46.30	49.07
35to64	38.57	45.63	42.22
65plus	9.39	8.07	8.71
Marital status			
Married	41.68	38.93	40.25
Single	58.32	61.07	59.75
Place of residence			
Urban formal	64.54	68.69	66.69
Urban informal	7.79	6.44	7.09
Tribal	23.51	21.91	22.69
Rural	4.16	2.96	3.54
Subjective social status			
Mean	4.56	4.14	4.35
S.D	2.12	2.06	2.10
Quintile 1 (rank 1–2)	21.56	27.19	24.47
Quintile 2 (rank 3–4)	24.62	27.01	25.86
Quintile 3 (rank 5–6)	36.49	33.52	34.96
Quintile 4 (rank 7–8)	13.88	10.13	11.94
Quintile 5 (rank 9–10)	3.44	2.15	2.77
Financial difficulties			
Hardly/Never	29.09	26.37	27.69
Always/Sometimes	70.91	73.63	72.31
Childhood conflict			
Hardly/Never	54.19	54.57	54.39
Always/Sometimes	45.81	45.43	45.61
Employment			
Employed	42.33	25.73	33.70
Non labour force participant	20.01	29.18	24.78
Unemployed	37.66	45.09	41.53
Education			
None	2.82	4.35	3.61
Primary	11.39	13.49	12.48
Secondary	38.42	37.17	37.77
Matric	36.31	35.42	35.85
Tertiary	11.07	9.57	10.30
Fruits			
< once a day	49.64	51.71	50.71
> = once a day	50.36	48.29	49.29
Vegetables			
< once a day	43.48	42.54	42.99
> = once a day	56.52	57.46	57.01
Physical activity			
0–2 times	44.11	59.12	51.75
3–7 times	55.89	40.88	48.25
Smoking			
Less than daily/never	68.37	91.24	80.19
Daily	31.63	8.76	19.81
Alcohol			
< =once a week/never	90.07	95.40	92.82
> once a week	9.93	4.60	7.18

More than 70% of adult respondents reported experiencing financial difficulties during childhood and 46% reported experiencing childhood conflict. Most of the participants were unemployed (42%) and had some secondary education (38%). With regards to lifestyle factors, approximately half of the participants consumed fruits less than once a day (51%), vegetables more than once a day (57%), exercised 0–2 times a week (52%), and never smoked or did not smoke daily (80%). Self-reported alcohol consumption was low. The majority of respondents never consumed alcohol or consumed it less than once a week (93%).

### Distribution of depressive symptoms by subjective social status

**Fig. 1:**
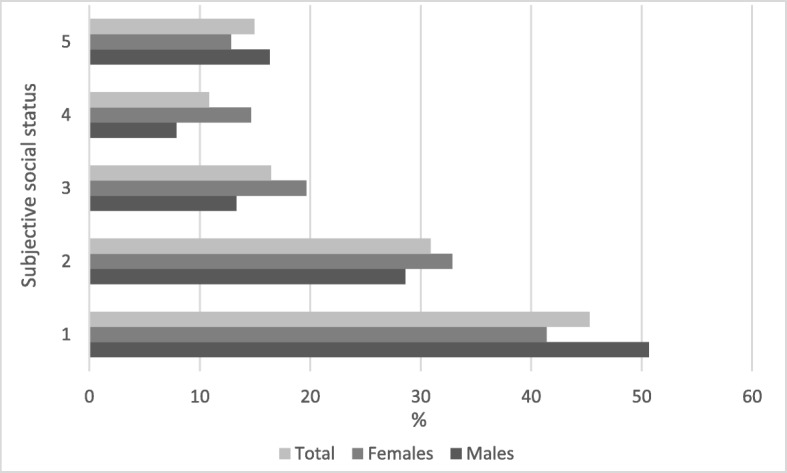
Prevalence of depressive symptoms, by subjective social status and gender

Figure 1 shows the prevalence of depressive symptoms by SSS and gender. The prevalence of depressive symptoms is highest in the lowest quintile, at 45%. The fourth quintile recorded the lowest prevalence of depressive symptoms, at 10%, before increasing slightly to 15% in the fifth quintile. Generally, there is a statistically significant relationship between the prevalence of depressive symptoms and SSS, for both men and women and on aggregate (*p* < 0.001). In all quintiles other than quintiles 1 and 5, the prevalence of depressive symptoms is highest among females when compared to males.

**Fig. 2:**
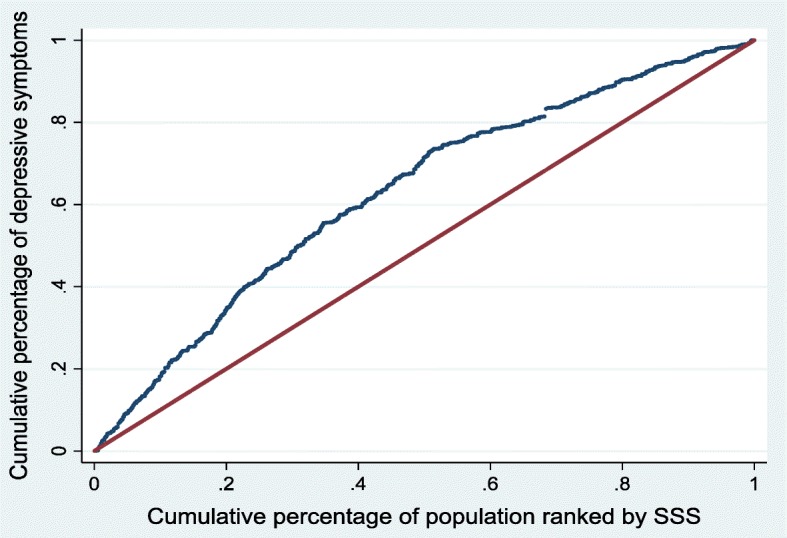
Concentration curve for depressive symptoms – overall

### Concentration curves and indices for depressive symptoms

**Fig. 3:**
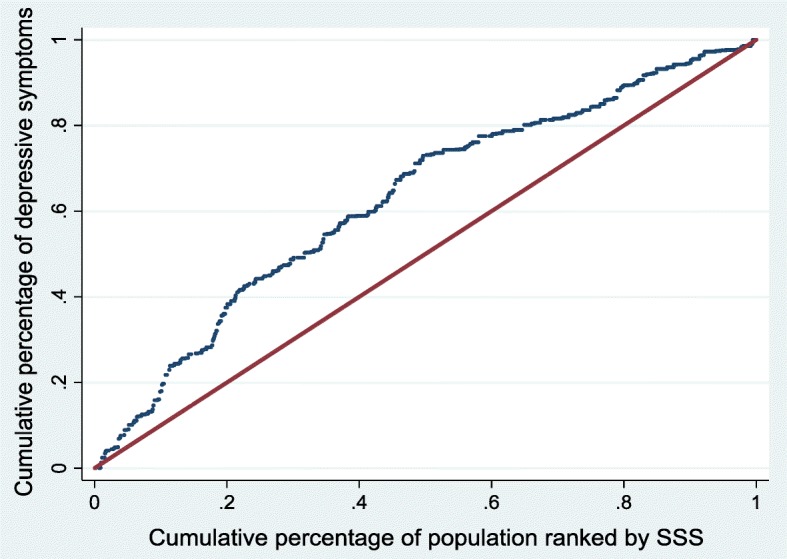
Concentration curve for depressive symptoms - males

**Fig. 4:**
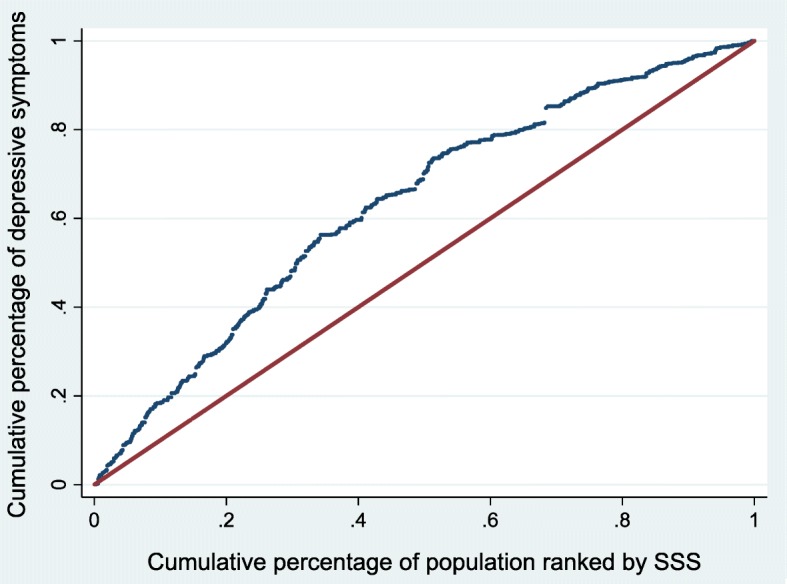
Concentration curve for depressive symptoms - females

Figures 2, 3 and 4 show the CC for the SSS-related inequalities in overall depressive symptoms, depressive symptoms amongst males and depressive symptoms amongst females, respectively. In all three instances, the curve lies above the 45-degree line of equality, showing that depressive symptoms are more concentrated amongst those with low SSS. All the CI are statistically significant and favour those with higher SSS for both males and females (i.e. ill health is concentrated among those of lower social status), as indicated by all curves lying above the line of equality. The observed SSS-related inequality in depressive symptoms is higher for males (CI = -0.304) when compared to females (CI = -0.240). This indicates that there is more inequality in the distribution of depressive symptoms among males when compared to females. The difference, however, is only weakly significant in statistical terms (*p* = 0.056)

### Decomposition of SSS-related inequalities in depressive symptoms

Results from the decomposition by gender are shown in Table 2. The first two columns show the elasticities and CI for each explanatory variable. The rest of the columns show the absolute contributions, their bootstrapped standard errors, the relative percentage contributions and the total percentage contributions of each explanatory variable to SSS-related inequalities in depressive symptoms.

**Table 2:** Decomposition of inequalities in depressive symptoms related to subjective social status, by gender

Variable	Male						Female						Total					
	Elasticity	CI	Abs	SE	%	Total	Elasticity	CI	Abs	SE	%	Total	Elasticity	CI	Abs	SE	%	Total
Gender																		
Female																		
Male													− 0.0152	0.0570	− 0.0035	0.0037	1.25	1.25
Race																		
African	*(base)*						*(base)*						*(base)*					
Coloured	−0.019	0.354	− 0.027	0.011	8.77		− 0.008	0.285	− 0.009	0.006	3.62		−0.011	0.318	−0.014	0.006	4.99	
Indian/Asian/White	−0.022	0.585	−0.052	0.022	17.15	25.92	0.001	0.602	0.003	0.018	−1.29	2.33	−0.007	0.593	−0.016	0.014	5.81	10.80
Age																		
18to34	*(base)*						*(base)*						*(base)*					
35to64	0.088	−0.025	− 0.009	0.010	2.89		0.025	−0.009	−0.001	0.003	0.35		0.048	−0.021	−0.004	0.004	1.43	
65plus	0.015	0.071	0.004	0.006	−1.35	1.54	0.002	−0.016	0.000	0.002	0.05	0.40	0.006	0.029	0.001	0.002	−0.26	1.18
Marital status																		
Married	*(base)*						*(base)*						*(base)*					
Single	0.087	−0.073	−0.026	0.010	8.41	8.41	0.021	−0.043	−0.004	0.004	1.48	1.48	0.039	−0.058	−0.009	0.005	3.24	3.24
Place of residence																		
Urban formal	*(base)*						*(base)*						*(base)*					
Urban informal	−0.002	−0.158	0.001	0.005	−0.45		0.003	−0.355	−0.004	0.008	1.69		0.001	−0.246	−0.001	0.004	0.30	
Tribal	−0.004	−0.196	0.003	0.008	−1.06		−0.012	−0.166	0.008	0.008	−3.38		−0.007	−0.178	0.005	0.005	−1.78	
Rural	−0.003	0.075	−0.001	0.005	0.34	−1.17	−0.003	−0.056	0.001	0.002	−0.32	−2.01	−0.003	0.025	0.000	0.002	0.10	−1.38
Subjective social status	−0.126	0.263	−0.133	0.048	43.65	43.65	−0.179	0.280	−0.201	0.049	83.46	83.46	−0.154	0.273	−0.168	0.038	60.97	60.97
Childhood financial difficulties																		
Hardly/Never	*(base)*						*(base)*						*(base)*					
Always/Sometimes	0.014	−0.132	−0.008	0.016	2.50	2.50	0.024	−0.096	−0.009	0.014	3.74	3.74	0.025	−0.114	−0.011	0.010	4.06	4.06
Childhood conflict																		
Hardly/Never	*(base)*						*(base)*						*(base)*					
Always/Sometimes	0.021	−0.142	−0.012	0.012	3.96	3.96	0.082	−0.124	−0.041	0.011	16.83	16.83	0.058	−0.132	−0.031	0.008	11.07	11.07
Employment																		
Employed	*(base)*						*(base)*						*(base)*					
Non labour force participant	−0.004	−0.027	0.000	0.003	−0.15		0.021	0.065	0.006	0.005	−2.32		0.010	0.019	0.001	0.002	−0.26	
Unemployed	0.011	−0.167	−0.007	0.010	2.36	2.21	0.000	−0.166	0.000	0.015	0.04	−2.27	0.004	−0.171	−0.003	0.011	0.89	0.63
Education																		
None	*(base)*						*(base)*						*(base)*					
Primary	0.003	−0.212	−0.003	0.009	0.87		−0.006	−0.236	0.005	0.012	−2.20		−0.005	−0.230	0.005	0.008	−1.77	
Secondary	−0.005	−0.160	0.003	0.022	−0.98		−0.007	− 0.084	0.002	0.012	−0.92		− 0.012	−0.120	0.006	0.011	−2.13	
Matric	−0.017	0.113	−0.008	0.015	2.48		0.004	0.097	0.002	0.013	−0.60		− 0.014	0.106	− 0.006	0.009	2.16	
Tertiary	−0.004	0.490	−0.008	0.024	2.73	5.10	−0.003	0.427	−0.006	0.019	2.27	−1.46	−0.007	0.460	−0.012	0.014	4.38	2.63
Fruits																		
< once a day	*(base)*						*(base)*						*(base)*					
> = once a day	0.016	0.090	0.006	0.008	−1.93	−1.93	−0.056	0.118	−0.027	0.011	11.00	11.00	−0.026	0.106	−0.011	0.007	3.99	3.99
Vegetables																		
< once a day	*(base)*						*(base)*						*(base)*					
> = once a day	−0.008	0.041	−0.001	0.004	0.42	0.42	0.042	0.084	0.014	0.009	−5.81	−5.81	0.021	0.063	0.005	0.005	−1.90	−1.90
Physical activity																		
0–2 times	*(base)*						*(base)*						*(base)*					
3–7 times	−0.002	0.000	0.000	0.002	0.00	0.00	−0.026	0.069	−0.007	0.005	2.97	2.97	−0.018	0.038	−0.003	0.003	0.99	0.99
Smoking																		
Less than daily/never	*(base)*						*(base)*						*(base)*					
Daily	0.004	−0.092	−0.002	0.005	0.48	0.48	0.022	0.183	0.016	0.004	−6.67	−6.67	0.010	0.002	0.000	0.002	−0.03	−0.03
Alcohol																		
< =once a week/never	*(base)*						*(base)*						*(base)*					
> once a week	0.001	−0.074	0.000	0.003	0.06	0.06	−0.010	−0.058	0.002	0.004	−0.95	−0.95	− 0.003	−0.050	0.001	0.002	−0.19	−0.19
*Explained*			−0.278			91.15			−0.248			103.06			−0.269			97.32
*Residual*			−0.027			8.85			0.007			−3.06			−0.007			2.68
*Total*			−0.304			100.00			−0.241			100.00			−0.276			100.00

Note: CI – Erreygers concentration index, Abs – Absolute contribution. SE- Bootstrapped standard errors for the absolute contributions obtained via a bootstrapping method using 500 replications. Residual is the part of the inequalities in depressive symptoms not explained by the chosen variables

As the table also shows, SSS makes the largest contributions to SSS-related inequalities in depressive symptoms (61%). This means that SSS-related inequalities in depressive symptoms would decrease by over 61% if there were no inequalities in SSS. It is notable that the contribution made by SSS to inequalities in depressive symptoms is much higher for females compared to males, 82% versus 44%. Other factors that make significant contributions to SSS-related inequalities in depressive symptoms are race and past childhood conflict. In the decomposition analyses, race makes the larger contribution to SSS-related inequalities in depressive symptoms for males compared to females (26% versus 2%). In contrast, past childhood conflict makes the larger contributions to SSS-related inequalities in depressive symptoms for females when compared to males (17% versus 4%). This means that SSS-related inequalities in depressive symptoms amongst females (males) would decrease by 17% (4%) if there were no inequalities in childhood adversity. In statistical terms, the other variables contribute only marginally to SSS-related inequalities in depressive symptoms. The lifestyle factors in particular make a relatively small contribution to SSS-related inequalities in depressive symptoms compared to other variables. The factors included in our study explained 97% (or 0.269) of the overall SSS-related inequality in the prevalence of depressive symptoms.

From the table we can also see that residing in informal urban settlements, having experienced financial difficulties or household conflict during childhood, being unemployed, and consuming alcohol more than once per week is more concentrated amongst people of low SSS as indicated by the negative CIs. In contrast, having a tertiary education, consuming fruits and vegetables more than once a day, and exercising more often, as expected is more concentrated amongst those of high SSS, as shown by the positive CIs.

## Discussion

At 26%, the overall prevalence of depressive symptoms is relatively high. This figure is comparable to the 2012 estimate of 27.1% from the National Income Dynamic Study (NIDS) that uses the CES-D 10’s cut-off score of 10 [30]. The estimate also falls within the wide although not directly comparable range of prevalence rates reported in other CES-D 10-based studies using the nationally representative NIDS. Pengpid et al. for example reported a prevalence rate of 13% for 2014/15 using a cut-off score of 12 [32], while Burns et al. reported a prevalence as high as 38.9% for 2008 using the cut-off score of 10 [31]. Similar to the significant gender difference reported here, other studies also found women to have an increased risk of depression [31, 33], higher CES-D 10 scores [30], and increased stress [34].

The one main objective of the paper was to quantify, using the CI, the extent of SSS-related inequality in depressive symptoms. Similar to evidence on self-reported health presented elsewhere [20, 21], the CIs for depressive symptoms in our study revealed an unequal distribution of depressive symptoms among South Africans. Our results likewise show significant SSS-related inequalities that favour those with higher SSS rank, with depressive status being concentrated in those of lower social status. Although our study makes use of SSS, our result is consistent with previous evidence from South Africa that used objective socio-economic status to measure inequalities in depression. These studies also report negative concentration indices, which shows disparities in depression that favour the better off [62, 63]. Various authors have also shown that low socio-economic status is a risk factor for mental health illness such as depression [30–32, 34, 64, 65]. Our results are also consistent with other studies that make use of the CI to measure SSS inequalities and find inequalities in self-rated health that favour the top social classes [20, 21]. In fact, the extent of inequality is much more pronounced in this developing country context than that reported in the only other comparable study conducted in a developed country, namely Spain [20]. In this study, the reported concentration indices, though negative as well, are as low as − 0.021 and − 0.045 respectively for the two measures of self-reported health status. Consistent with other studies that also make use of the CES-D scale, we also find that the prevalence of depressive symptoms was higher in females when compared to males [30, 32].

Our paper also sought to examine the contribution of various factors to SSS-related inequalities in depressive symptoms both on aggregate and by gender. Using a decomposition approach, we find that SSS, race and past childhood adversity make important contributions to SSS-related inequalities in depressive symptoms. Our results are in line with previous studies that found that SSS has a significant influence on depression [5, 12, 13, 15, 34, 50]. Using RII, studies by Adler at al. and Miyakawa et al. both show a significant relationship between depression and SSS [12, 13]. Of note in this study was the difference in the contributions of SSS across gender. The contribution of SSS to inequalities in depressive symptoms was higher for women than men. This highlights the importance of considering the unique ways in which men and women conceptualise and evaluate their place in the social hierarchy when interpreting the impact of SSS on depression. As noted in Shaked et al., the factors that may be pertinent in predicting SSS for women differ to those for men [66] and thus the strengths of association would differ, as is the case in this particular study.

The contribution of the childhood adversity variable to SSS-related inequalities was also higher for females when compared to males. Other studies also point to the effect of childhood adversities on mental health [26, 54], with stronger associations being recorded for females when compared to males [67]. This may be attributable to the differences in the psychology of emotions in women versus men or the different influences of specific types of childhood adversities in women versus males. Findings from a study by Veijola et al. show that more childhood factors were associated with depression in females when compared to males [68].

Race was another variable that showed some influence on inequalities in depressive symptoms. The relationship in South Africa between race and the prevalence of illness related to depression has been documented before [30, 31, 65]. In South Africa, the race variable is intricately connected with social status, which varies due to the direct and indirect effects of the discriminatory socioeconomic policies under the apartheid system. This makes it difficult to separate the associations of race and depressive symptoms from the associations of SSS with depressive symptoms. The contribution of race to inequalities in the prevalence of depressive symptoms related to SSS also points to the importance of cultural differences in shaping the expression and experiences of depression [69]. Our study further shows that the contribution of race to SSS inequalities in depressive symptoms is higher amongst males when compared this females. Such differences in contribution may be due to the effect of race on inequalities in depressive symptoms amongst females being captured by other significant variables such as SSS.

This study has several limitations. Firstly, the use of a cross sectional dataset limits the discussion of causal relationships between SSS-related inequalities and its various determinants, with the result that evidence points to associations only. In future studies questions of causality may be addressed by use of longitudinal data. Secondly, the study uses a self-reported measure of depression, rather than clinical diagnosis, and also relies on self-report for the measurement of some covariates included in the analysis, which may introduce some subjectivity and bias into the results. This study, furthermore, employs a broad societal reference group in its operationalisation of the SSS construct. Using closer referents such as community members or similar others, which may be more appropriate in regards to mental health, may yield different results [70–72]. Further, our study does not present information on the distribution of depressive symptoms in subjects below the age of 18 years. Future studies that cover this age group could better represent the contribution of SSS to overall inequalities in depression. Despite these caveats this paper has a major strength. The study makes use of nationally representative data to estimate SSS-related inequalities in depressive symptoms among South African adults. The analytical strategy combines the rigour of the European Social Survey’s approach to the study of the social determinants of health with information on subjective social status, which was not included in the standard ESS7 survey module.

## Conclusion

The results of this study document the presence of much more significant SSS-related inequalities in depressive symptoms favouring those of higher social status when compared to developed countries. To the best of our knowledge, this is the first study of its kind to apply decomposition analysis to the investigation of SSS-related inequalities in health. The study shows that the observed SSS-related inequalities in depressive symptoms are mostly explained by SSS itself followed by race and past childhood conflict. The size of the contributions made by these factors differ by gender, highlighting the importance of gender-based analysis in studies of health inequalities. Results from this study suggest that policy makers seeking to reduce SSS-related inequalities in depression should target a reduction in the positive contribution of SSS to depression via the implementation of programmes that improve social welfare. Given the much greater contribution to inequalities among females, these policies should target women. Policies that protect children and especially the girl child from conflict can also be useful in reducing inequalities in depression related to subjective social status during adulthood. Overall, there is need for a multi-sectoral approach to address these inequalities.

## Data Availability

The dataset(s) used in this article is available on request from the HSRC.

## Abbreviations

CI: Concentration indices
GHS: General Household Surveys
GLM: Generalised linear models
HSRC: Human Sciences Research Council
NIDS: National Income Dynamics Study
OLS: Ordinary least squares
SASAS: South African Social Attitudes Survey
SD: Standard deviation
SDG: Sustainable development goal
SSS: Subjective social status
